# Preparation of Type-A Gelatin/Poly-γ-Glutamic Acid Nanoparticles for Enhancing the Stability and Bioavailability of (-)-Epigallocatechin Gallate

**DOI:** 10.3390/foods12091748

**Published:** 2023-04-23

**Authors:** Weijie Zhang, Huangchen Shen, Ying Li, Kai Yang, Peng Lei, Yian Gu, Liang Sun, Hong Xu, Rui Wang

**Affiliations:** College of Food Science and Light Industry, State Key Laboratory of Materials-Oriented Chemical Engineering, Nanjing Tech University, No. 30 Puzhu South Road, Nanjing 211816, China

**Keywords:** type-A gelatin, γ-PGA, EGCG, nanoparticles, stability, bioavailability

## Abstract

(-)-Epigallocatechin gallate (EGCG) has gained considerable attention owing to its beneficial properties. However, its application as a functional food is restricted due to its instability and low bioavailability. In the present study, a food-derived nanoparticle system based on type A gelatin/γ-PGA was developed to preserve and deliver EGCG. The EGCG/gelatin/γ-PGA nanoparticles had a particle size of 155.1 ± 7.3 nm with a zeta potential of −23.9 ± 0.9 mV. Moreover, the EGCG/gelatin/γ-PGA nanoparticles enhanced the long-term storage stability and sustained antioxidant activity of EGCG compared to EGCG/gelatin nanoparticles. The nanoparticles protected EGCG in simulated gastric fluid containing pepsin while releasing it in simulated intestinal fluid. Additionally, the amount of EGCG transported in the Caco-2 monolayers treated with EGCG/gelatin/γ-PGA nanoparticles was three times higher than that of free EGCG, which might be related to the paracellular pathway and endocytosis. These results suggest that EGCG/gelatin/γ-PGA nanoparticles might be an effective delivery vehicle for EGCG, enhancing its potential applications in the functional food field.

## 1. Introduction

(-)-Epigallocatechin gallate (EGCG) is the most prevalent catechin component in green tea [1]. EGCG has strong oxidation resistance due to the substantial number of phenolic hydroxyl groups in its structure [2]. It has been demonstrated that EGCG regulates metabolism in the human body [3] and has anti-tumor [4] and anti-cardiovascular disease properties [5]. Because of these health advantages, it has drawn special interest in recent years and is considered a potential alternative to synthetic food additives [6] or commercial high-dose food supplements [7]. However, practical applications of EGCG are limited by several factors. First, EGCG is less resilient to temperature, light, pH, and oxygen, which significantly accelerates its breakdown during long-term storage [8]. Additionally, EGCG may be destroyed by digestive enzymes and other biologically active substances in gastrointestinal fluids after ingestion due to its hostile environment in humans [9]. Furthermore, the intestine prolongs the residence time of EGCG, resulting in insufficient EGCG permeability [10]. These defects significantly reduce the stability and bioavailability of EGCG, severely limiting its potential applications [11]. Therefore, an efficient EGCG delivery system is required.

Small intestinal epithelial cells can be penetrated by nanoparticles through either paracellular or endocytic pathways [12,13]. Therefore, the development of EGCG-loaded nanocarriers is crucial for overcoming the instability and low bioavailability of EGCG. EGCG has been encapsulated in multiple nanodelivery systems [14,15], including nanoparticles [16] and nanoemulsions [17]. Nanoparticles with proteins and polysaccharides as wall materials have been widely used in the production of nanocarrier systems due to their high biocompatibility, degradability, and biosafety [18]. Under specific pH conditions, proteins and polysaccharides form nanocomplexes via various interactions to encapsulate and control the release of EGCG. Complexes such as zein/caseinate [19], γ-PGA/chitosan [12], and whey protein/cellulose [20] have been used for EGCG encapsulation.

Poly γ-glutamic acid (γ-PGA) is an anionic polypeptide polymer produced through microbial fermentation and possesses edible and beneficial properties [21]. It is highly hydrophilic due to the ionization of side-chain carboxyl groups in an aqueous solution. γ-PGA has been widely employed as a carrier material in various fields, including cosmetics, agriculture, food, and drug delivery systems [22]. Studies have shown that γ-PGA specifically enhances the intestinal absorption of low molecular weight functional components, such as vitamins, polyphenols, carotenoids, and physiologically active peptides [23]. Additionally, γ-PGA exhibits biological activity in glucose and lipid metabolism as a food additive [24]. It has been reported that γ-PGA can improve the antihyperlipidemic activity of isoflavones through combination administration [25]. 

Gelatin, a protein derived from the partial hydrolysis of naturally existing collagen, contains free amino and carboxyl groups in its structure [26]. Gelatin forms thermoreversible hydrogels that have been utilized in the food and pharmaceutical industries to develop microcapsules or nanocarriers for specific drug delivery [27,28]. As a polyampholyte, gelatin forms complexes with either cationic or anionic polymers under different pH conditions. Type-A gelatin is produced through partial hydrolysis of collagen with acid, while type-B gelatin is produced through alkaline hydrolysis [29]. Compared to type-B gelatin (pI = 4–5), type-A gelatin (pI = 7–9) has a positive charge and can interact with negatively charged EGCG under weakly alkaline pH conditions, which has been used for EGCG encapsulation [26,30]. Several studies have reported on the potential of delivery systems based on gelatin/γ-PGA hydrogel [31,32]. However, the impacts of gelatin/γ-PGA nanoparticle encapsulation on EGCG storage and delivery have not been investigated.

In this study, we used type-A gelatin and γ-PGA to self-assemble complex nanoparticles (EGCG/gelatin/γ-PGA) for encapsulating and delivering EGCG. We investigated the impact of preparation conditions on the physical properties of the nanoparticles and obtained optimal EGCG/gelatin/γ-PGA nanoparticles. The feasibility of the nanoparticles as EGCG carriers was confirmed using a series of assays. We found that EGCG/gelatin/γ-PGA nanoparticles improved the stability and antioxidant ability of EGCG more effectively than EGCG/gelatin nanoparticles. Moreover, EGCG/gelatin/γ-PGA nanoparticles successfully preserved EGCG in simulated gastric fluid with low pH and pepsin and increased the permeability of Ca-co-2 monolayers. This study presents a novel delivery system for EGCG in the food industry.

## 2. Materials and Methods

### 2.1. Materials

Gelatin (type-A, pI = 8.0) and EGCG (purity ≥ 98%, HPLC) were purchased from Yuanye Bio-Technology Co., Ltd. (Shanghai, China). Poly γ-glutamic acid (γ-PGA; MW = 600 kDa) was obtained from Nanjing Shineking Biotech Co., Ltd. (Nanjing, China). EGCG (purity ≥ 90%) was kindly supplied by Anhui Agricultural University (Anhui, China). Simulated gastric fluid (SGF; pH 2.1 with pepsin) and simulated intestinal fluid (SIF; pH 7.2, with trypsin) were purchased from Yuanye Microbes (Shanghai, China). 1,1-Diphenyl-2-picrylhydrazyl (DPPH) and DPPH free radical scavenging capacity assay kits were purchased from Nanjing Jiancheng Bioengineering Institute (A153-1-1, Nanjing, China). The total antioxidant capacity assay kit using the ABTS method was purchased from the Beyotime Institute of Biotechnology (S0119, Nantong, China). Caco-2 (human colorectal cancer cells) lines were purchased from ProCell Life Science and Technology Co., Ltd. (Wuhan, China), while the Caco-2 cell-specific medium was purchased from Yifeixue Biotechnology (Nanjing, China). 

### 2.2. Preparation of Complex Nanoparticles

The preparation of the EGCG/gelatin nanoparticles was based on an experiment by Chen et al. (2010) with slight modifications [33]. Briefly, aqueous solutions of gelatin (1.0, 2.0, 4.0, 6.0, and 8.0 mg/mL, 1 mL, pH 6.2) were added with a simple injection device into EGCG solutions (0.5, 1.0, 2.0, 3.0, and 4.0 mg/mL, 1 mL, in deionized water) and stirred (200 r/min) for 2 h at room temperature (25 °C). EGCG/gelatin nanoparticle suspensions were used for further analyses and applications. To prepare the EGCG/gelatin/γ-PGA nanoparticles, an aqueous solution of EGCG (0.5, 1.0, 2.0, 3.0, and 4.0 mg/mL, 1 mL) was mixed with the γ-PGA solution (1.0 mg/mL, 1 mL, pH 6.2) before adding the gelatin solution (1.0, 2.0, 4.0, 6.0, and 8.0 mg/mL, 1 mL, pH 6.2). The obtained EGCG/gelatin/γ-PGA nanoparticle suspensions were tested in the same manner. 

### 2.3. Characterization of the Produced Nanoparticles

The particle size distribution, polydispersity index (PDI), and zeta potential of the nanoparticles were measured using a Zetasizer Nano (Malvern ZS90, UK) and dynamic light scattering (DLS) techniques. Fourier-transform infrared (FT-IR) spectroscopy was used to analyze the chemical structures of EGCG, type-A gelatin, γ-PGA, and the nanoparticles within the range of 500 to 4000 cm^−1^.

The fluorescence spectra of the nanoparticles were recorded using a fluorescence spectrophotometer at two different temperatures (25 °C and 37 °C). The nanoparticle solution was diluted ten times with phosphate buffer (pH 6). The excitation wavelength was set to 280 nm, and the emission range was 300–500 nm, with both excitation and emission slit widths set to 5 nm.

The apparent morphology of the two types of nanoparticles was confirmed using scanning electron microscopy (SEM) and transmission electron microscopy (TEM). The SEM samples were freeze-dried for 48 h and sputter-coated with gold for 120 s before observation (Hitachi S4800, Tokyo, Japan). The TEM samples were prepared by depositing a drop of the nanoparticle suspension onto a carbon-coated copper grid with a mesh size of 400, followed by removing surface water using filter paper and drying with air. The dried samples were then examined using transmission electron microscopy (TEM) (Hitachi H-600, Japan).

### 2.4. Assessments of Encapsulation Efficiency of EGCG in Nanoparticles

The prepared small-particle-size nanoparticles were collected via high-speed centrifugation (15,000 rpm, 4 °C, 15 min). The supernatant was then filtered through a 0.22 μm membrane, and the amount of free EGCG was assessed via high-performance liquid chromatography (HPLC) [34]. Briefly, an Agilent 1260 HPLC system (Agilent Technologies Inc., Santa Clara, CA, USA) equipped with a UV detector (SPD-20A) and a Sepax GP-C18 column (4.6 mm × 250 mm) was utilized. The mobile phase consisted of ddH_2_O, ACN, and TFA (919/80/1, *v/v*) for Phase A and ddH_2_O, ACN, MeOH, and TFA (699/270/30/1, *v/v*) for Phase B with a system flow rate of 1.2 mL/min. The injection volume was 10 μL, and the column oven temperature was set to 25 °C. Gradient elution was performed as follows: 95/5 at 0 min, 30/70 at 1.5 min (convex), 1/99 at 3 min (convex), and 95/5 at 3–5 min (step immediate). The total chromatographic runtime was 9.5 min. Individual EGCG content was quantified by constructing multilevel calibration curves (EGCG concentrations: 25, 50, 75, 100, 150, and 200 ppm) from the response at 280 nm caused by the injection of authentic EGCG standards. Total EGCG was computed as the sum of all individual EGCG measurements. The EGCG encapsulation efficiency (EE) and loading capacity (LC) of the nanoparticles were calculated using the following formulas:

(1) $$Encapsulation\;efficiency\;(\%)\;=\;(E_{1}\;-\;E_{2})/E_{1}$$

(2) $$Loading\;capacity\;(\%)\;=\;(E_{1}\;-\;E_{2})/E_{3}$$

where *E*_1_ and *E*_2_ are the total amounts of EGCG and free EGCG as determined by HPLC, respectively, and *E*_3_ is the weight of the nanoparticles.

### 2.5. Storage Stability of EGCG/Gelatin/γ-PGA Nanoparticles

The nanoparticle suspensions were stored in a dark place at 4 °C and 25 °C for 28 d. The particle size and PDI of the nanoparticles were measured every 7 d. Before testing for nanoparticle dispersion, each sample was ultrasonicated for 1 min at 20 kHz and 400 W. The experiments were performed in triplicate.

### 2.6. EGCG Release of Nanoparticles under Different pH Conditions

To investigate the pH sensitivity of the nanoparticles, they were dispersed in buffer solutions with varying pH values. First, the EGCG/gelatin/γ-PGA nanoparticle suspensions were treated with high-speed centrifugation (15,000 rpm, 4 °C, 15 min), and the sediments were then ultrasonically dispersed in buffer solutions. The pH was adjusted to 3.0, 4.0, 5.0, 6.0, 7.0, and 7.5 using 0.1 M HCl or NaOH solution. The EGCG/gelatin/γ-PGA nanoparticle suspensions were then incubated at 37 °C in a water bath for 2 h. After incubation, 2.0 mL of the sample suspensions were collected and centrifuged to obtain the supernatant. The amount of EGCG released was evaluated using HPLC by measuring the EGCG content in the supernatant. The percentage decline in the EGCG content of the nanoparticles was used to calculate the amount of EGCG released after centrifugation. All experiments were performed in triplicate.

(3) $$EGCG\;Release\;(\%)\;=\;(E_{1}\;-\;E_{2})/E_{1}$$

where *E*_1_ and *E*_2_ represent the total amounts of EGCG and free EGCG, respectively.

### 2.7. In Vitro Release of the Complex Nanoparticles

In vitro simulated digestion experiments were conducted using simulated gastric fluid (SGF, pH 2.1, with pepsin) and simulated intestinal fluid (SIF, pH 7.2, with trypsin). The nanoparticles were incubated in SGF and SIF for 0–4 h, and the amount of EGCG released was measured using HPLC. The centrifuged nanoparticles were dispersed in SGF or SIF (0 h) and magnetically stirred (200 rpm) in a 37 °C water bath. The amount of EGCG in the supernatant was quantified at various time points (1, 2, 3, and 4 h) using HPLC. 

### 2.8. Antioxidant Measurements of Complex Nanoparticles by DPPH/ABTs

The free radical scavenging activity of the EGCG/gelatin and EGCG/gelatin/γ-PGA nanoparticles against DPPH was measured using the DPPH scavenging assay kit [35]. Briefly, 0.4 mL of the sample solution was mixed with 0.6 mL of DPPH working solution and left in the dark at 25 °C for 30 min before centrifugation at 4000 rpm for 5 min. The absorbance of the reaction solution was measured at 517 nm using an enzyme-labeled instrument. The DPPH radical scavenging activities of the different samples were calculated using the following formula:

(4) $$DPPH\;radical\;scavenging\;activity\;\left(\%\right)\;=\;[1\;-\;\left(A_{s}\;-\;A_{c}\right)/A_{b}]\;\times \;100\%$$

Here, *A_c_* represents the absorbance of the control (sample solution mixed with 80% methanol), *A_s_* represents the absorbance of the sample (sample solution mixed with working solution), and *A_b_* represents the absorbance of the blank (80% methanol mixed with working solution).

To assess ABTS radical scavenging activity, an ABTS total antioxidant capacity test kit was used [36]. Firstly, the ABTS solution in the kit was mixed with an equal volume of oxidant solution and allowed to react for 16 h at 25 °C in the dark to prepare the ABTS working solution. Next, the ABTS working solution (1 mL) was mixed with 80% ethanol (99 mL) to obtain a fresh ABTS solution with an absorbance of approximately 0.7 at 734 nm. Then, the sample solution (10 μL) and fresh ABTS solution (200 μL) were added sequentially and reacted at 25 °C for 6 min. The absorbance of the samples was immediately measured at 734 nm. The ABTS radical scavenging activity of the samples was calculated using the following formula:

(5) $$ABTS\;radical\;scavenging\;activity\;(\%)\;=\;((A_{b}\;-\;A_{s})/A_{b})\;\times \;100\%$$

where *A_s_* represents the absorbance of the sample at 734 nm and *A_b_* represents the blank (distilled water) absorbance at 734 nm.

### 2.9. EGCG Transportation on Caco-2 Cell Monolayers

Caco-2 cells were cultured on 12-well transwell plates (Corning Inc., Corning, NY, USA) with 0.4 μm aperture, 12 mm inner diameter, and 1.12 cm^2^ growth area. The seeding density was 3 × 10^5^ cells, and the cells were maintained in Caco-2 cell-specific medium (Yifeixue) in the donor and receiver compartments to promote cell growth. The medium was changed every 48 h for the first week and every 24 h thereafter. The cells were incubated at 37 °C with 95% air and 5% CO_2_ for 18–21 days, and the transepithelial electrical resistance (TEER) was measured to be over 400 Ω cm^2^ before conducting the experiments.

The transportation study was conducted with minor adjustments to a previously published protocol [37]. TEER values of the Caco-2 monolayers were measured using a Millipore Millicell ERS-2 Cell Resistor (Millipore Corp., Burlington, MA, USA). The transport medium for permeation experiments was HBSS (pH 6.2). Before the experiment, Caco-2 monolayers were rinsed twice and adaptively cultured for 30 min in the transport medium. Free EGCG, EGCG/gelatin nanoparticles, and EGCG/gelatin/γ-PGA nanoparticles were added as the donor medium to maintain the EGCG concentration at 0.025 mg/mL. Caco-2 monolayers were then treated with the test solutions (0.5 mL) in the donor compartment and 1.5 mL of the transport medium (HBSS, pH 6.2) in the receiver compartment. The control group was treated with equal volumes of HBSS in the donor compartment. The plates were incubated on a shaker at 150 rpm at 37 °C. Samples (50 μL) were collected from the receiving compartment at different time intervals (30, 60, 90, and 120 min), and HPLC analysis was used to measure the EGCG concentrations [34]. The cumulative transport rate of EGCG was calculated and plotted against time.

The integrity of the Caco-2 monolayers was maintained by measuring the TEER value before each experiment, which was calculated as follows:

(6) $$TEER\left(\Omega {cm}^{2}\right)\;=\;\left(TEER\left(\Omega \right)\;-\;{TEER}_{b}\left(\Omega \right)\right)\;\times \;Area\left({cm}^{2}\right)$$

where *TEER* (Ω) is the transmembrane resistance of Caco-2 monolayers, read directly from the voltmeter, *TEER_b_* (Ω) is the background that transwell plates (without cells) supplemented with Caco-2 cell-specific medium. The area (cm^2^) was the membrane area of the transwell plates, 1.12 cm^2^.

### 2.10. Statistical Analysis

All experiments were conducted in triplicate. Single-way analysis of variance (ANOVA) using the Statistical Package for the Social Sciences (SPSS) version 18.0 (Chenna Inc., Chicago, IL, USA) was performed to examine the variations in samples, with a significance level of α = 0.05. Post hoc tests were conducted where appropriate. Data are presented as means ± standard deviations (SDs).

## 3. Results and Discussion

### 3.1. Preparation of Complex Nanoparticles

The use of gelatin and γ-PGA for the preparation of EGCG/gelatin/γ-PGA nanoparticles was based on their food safety and biocompatibility. The formation of nano complexes was achieved through the electrostatic interaction between γ-PGA and gelatin, while stable EGCG nanoparticles were formed via hydrogen bonding with gelatin [33]. To ensure the stability and optimal biological properties of the nanoparticles, it is important to investigate the impact of their physicochemical characteristics, particularly the mass ratio of their components, on the final product. Therefore, in this study, we evaluated the effect of the mass ratio of the components on the physical properties of EGCG/gelatin/γ-PGA nanoparticles.

Initially, we investigated the characteristics of nanoparticles that were prepared using different gelatin/γ-PGA mass ratios, ranging from 1:1 to 8:1, as presented in Table 1. The levels of EGCG and γ-PGA were maintained at 1 mg/mL throughout the study. With increasing concentrations of gelatin, the size and polydispersity index (PDI) of gelatin/γ-PGA nanoparticles gradually increased. Above a gelatin concentration of 6 mg/mL, we observed aggregation and precipitation of nanoparticles. This outcome is likely due to gelatin neutralizing the negative surface charge of the nanoparticles, thereby reducing their physical stability. Furthermore, the average particle size of EGCG/gelatin/γ-PGA nanoparticles was larger, and their absolute zeta potential was lower than that of gelatin/γ-PGA nanoparticles, indicating that EGCG was not simply embedded within the nanoparticles. All the nanoparticles had negative zeta potentials, and this is likely due to the phenol hydroxyl group of EGCG being slightly deprotonated at pH 6.2 [38] and the side-chain carboxylate group of γ-PGA being more ionized in weak acid solution because its pKa value is near 2.5 [39]. This is the primary reason for the negative charges of EGCG/gelatin/γ-PGA nanoparticles. Zeta potential has long been considered a reliable indicator for evaluating the stability of colloids [40]. In general, nanoparticles with zeta potentials above +30 mV or below −30 mV are deemed stable [41]. The EGCG/gelatin/γ-PGA nanoparticles had a higher absolute zeta potential than the EGCG/gelatin nanoparticles, indicating their greater stability in suspension [42].

**Figure 1:**
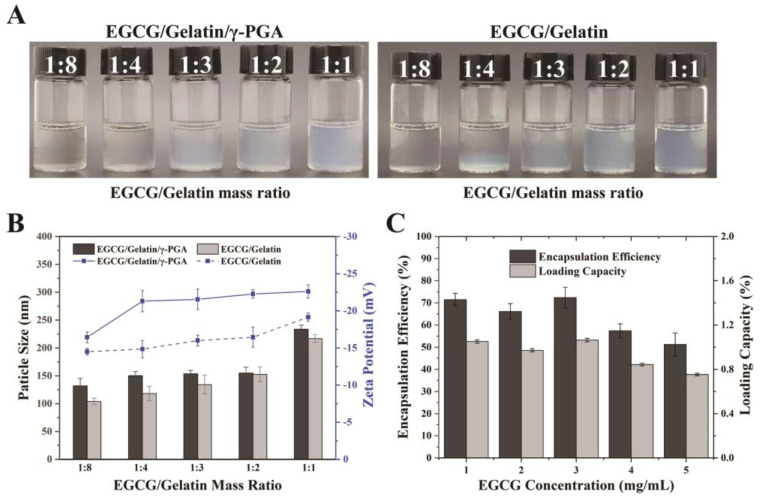
(**A**) The appearance of nanoparticle solutions prepared with varying EGCG/gelatin mass ratio; (**B**) Particle size and zeta potential of complex nanoparticles prepared with different EGCG concentrations; (**C**) Effects of EGCG concentrations on binding efficiency of EGCG/gelatin/γ-PGA nanoparticles.

Based on the previous experiments, the mass ratio of gelatin/γ-PGA at 4:1 was found to produce nanoparticles with favorable properties. Hence, the concentrations of gelatin and γ-PGA were fixed at 4 mg/mL and 1 mg/mL, respectively, and mixed with EGCG solutions of different concentrations. The mixture became increasingly turbid as the EGCG concentration increased (Figure 1A). The particle size and zeta potential of nanoparticles produced with varying EGCG/gelatin mass ratios are shown in Figure 1B. It was observed that EGCG concentration had little effect on the nanoparticles, and they maintained particle sizes of approximately 150 nm and an absolute zeta potential of 23 mV in most cases. However, when the EGCG/gelatin mass ratio was 1:1, the size of EGCG/gelatin/γ-PGA nanoparticles increased to 233 nm. Similarly, EGCG/gelatin/γ-PGA nanoparticles had larger particle sizes and absolute zeta potentials compared to EGCG/gelatin nanoparticles. While the majority of EGCG was located inside the nanoparticles, some may have bound to gelatin at the surface, affecting the potential of the nanoparticles. The EGCG/gelatin nanoparticles had a negative charge of zeta potentials at pH 6.2, and the absolute zeta potential decreased with increasing EGCG concentration, as the pKa value of EGCG was approximately 7.68. The encapsulation efficiency (EE) and loading capacity (LC) of EGCG/gelatin/γ-PGA nanoparticles were between 51.2–72.4% and 0.75–1.06%, respectively (Figure 1C). The highest EE was observed when the EGCG concentration was 2 mg/mL. However, as the concentration of EGCG mixed with the γ-PGA aqueous solution increased, the EE and LC of EGCG decreased. This was because both the EGCG content in the original mixture and the EGCG concentration outside the nanoparticles had increased. The encapsulation efficiency of EGCG is influenced by the partition between the nanoparticle space and the surrounding aqueous environment [43]. The high hydrophilicity of γ-PGA may explain the increase in encapsulation efficiency of EGCG/gelatin/γ-PGA nanoparticles. 

### 3.2. Characterization of Complex Nanoparticles

**Figure 2:**
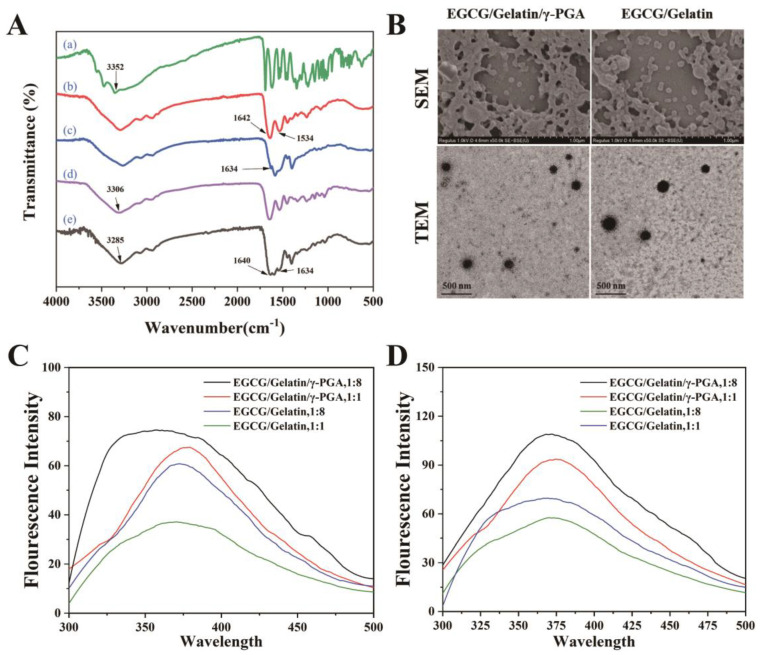
(**A**) FT-IR spectra of EGCG (a), gelatin (b), γ-PGA, (c) powder, and EGCG/gelatin nanoparticles (d), EGCG/gelatin/γ-PGA nanoparticles (e); (**B**) SEM and TEM micrography of EGCG/gelatin nanoparticles and EGCG/gelatin/γ-PGA nanoparticles. Fluorescence spectra of EGCG/gelatin and EGCG/gelatin/γ-PGA nanoparticles with different EGCG/gelatin mass ratios at 25 °C (**C**) and 37 °C (**D**).

The EGCG/gelatin/γ-PGA nanoparticles were produced by mixing a solution of γ-PGA (1 mg/mL) and EGCG (2 mg/mL) with a solution of type-A gelatin (4 mg/mL) at room temperature (25 °C) while stirring. The FT-IR absorption spectrums of EGCG, gelatin, γ-PGA, EGCG/gelatin nanoparticles, and EGCG/gelatin/γ-PGA nanoparticles were shown in Figure 2A. The absorption band at approximately 3352 cm^−1^ in EGCG was due to the vibrating O-H bond of the phenolic hydroxyl group. Gelatin showed strong amide absorptions, with the amide I C=O stretching band at 1642 cm^−1^ and the amide II N-H bending band at 1534 cm^−1^ [44]. γ-PGA exhibited strong absorption peaks at 1634 cm^−1^ due to the carboxyl group ion (-COO^−^) [12]. In the spectra of the gelatin/EGCG nanoparticles, the O-H peak of the phenolic hydroxyl group at 3352 cm^−1^ was red-shifted to 3306 cm^−1^ and became broader than that of EGCG. Other shifts in the characteristic peaks of EGCG/gelatin/γ-PGA nanoparticles, such as 3285 cm^−1^, 1541 cm^−1^, and 1640 cm^−1^, were also found, attributed to the phenolic hydroxyl of EGCG, the amide II of gelatin, and the carboxyl group of γ-PGA. These results suggested that the formation of nanoparticles was induced by electrostatic interactions and hydrogen bonding.

The morphology of the nanoparticles was analyzed using SEM and TEM techniques (Figure 2B). SEM images demonstrated that both types of nanoparticles were spherical in shape, with some adhesion between them, possibly due to the interaction between gelatin, EGCG, and γ-PGA. TEM results showed that the EGCG/gelatin/γ-PGA nanoparticles had a larger particle size than EGCG/gelatin nanoparticles, which was consistent with the particle size measurements obtained earlier. These findings confirmed the successful encapsulation of EGCG in the gelatin/γ-PGA nanoparticles. 

Fluorescence spectroscopy was used to investigate the interactions between gelatin, EGCG, and γ-PGA at different temperatures (Figure 2C,D). Gelatin fluorescence is mainly attributed to the intrinsic Tyr fluorophore, while Phe cannot be excited in most cases [45]. Additionally, γ-PGA containing only glutamic acid does not exhibit fluorescence. The EGCG/gelatin/γ-PGA nanoparticles were red-shifted as the gelatin/γ-PGA mass ratio changed from 1:8 to 1:1 at 25 °C, indicating that EGCG bound to the protein and changed its structure. The maximum emission wavelength of the EGCG/gelatin nanoparticles remained constant, indicating that there were no significant changes in the protein conformation. The fluorescence intensity of EGCG/gelatin/γ-PGA nanoparticles was higher than that of EGCG/gelatin nanoparticles at both 25 °C and 37 °C, and the fluorescence intensity of nanoparticles decreased with increasing EGCG concentration, which could be attributed to the fluorescence quenching of the interaction between Trp and EGCG. These findings suggest that EGCG interacted with gelatin in the nanoparticles, resulting in a reduction in fluorescence intensity.

### 3.3. Storage Stability of EGCG/gelatin/γ-PGA Nanoparticles

**Figure 3:**
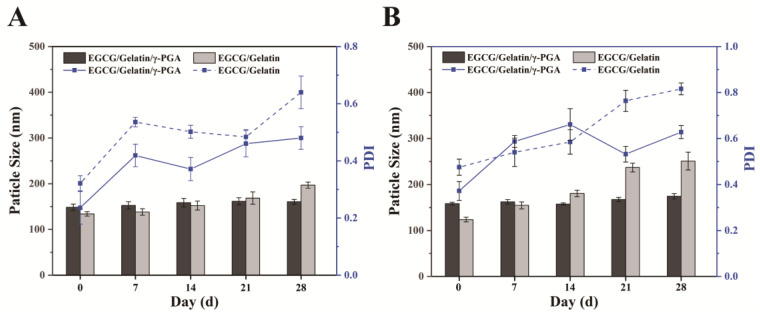
The effects of storage time on the particle size and PDI of EGCG/gelatin/γ-PGA nanoparticles at 4 °C (**A**) and 25 °C (**B**).

Despite the promising therapeutic effects of EGCG, its poor stability has hindered its large-scale production and use in the food industry. The effect of temperature on the stability of nanoparticles during storage was investigated (Figure 3). The particle size of EGCG/gelatin/γ-PGA nanoparticles remained constant throughout the storage period, with an average particle size of approximately 160 nm after 28 days. The PDI was maintained between 0.2 and 0.6, indicating that the EGCG/gelatin/γ-PGA nanoparticles were stable and did not significantly aggregate. The particle size and PDI of the EGCG/gelatin nanoparticles slowly increased during storage, which could be attributed to nanoparticle aggregation and swelling in the solution. Additionally, all nanoparticles exhibited lower PDI at 4 °C, and the average particle size of EGCG/gelatin nanoparticles was 196.9 nm, which was smaller than the 250.9 nm at 25 °C. These findings suggest that the EGCG/gelatin/γ-PGA nanoparticles have excellent stability for long-term storage at 4 °C.

### 3.4. In Vitro Bioaccessibility of EGCG in EGCG/Gelatin/γ-PGA Nanoparticles

**Figure 4:**
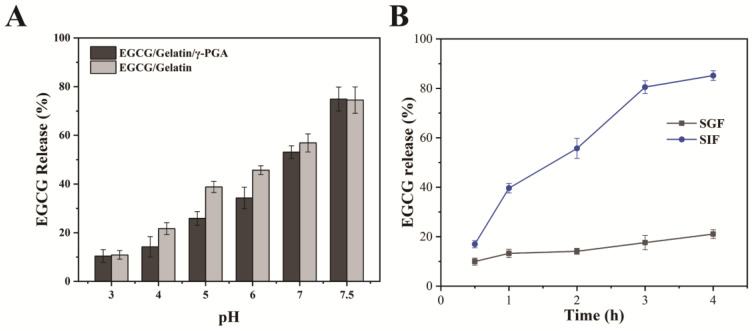
(**A**) EGCG release of the nanoparticles after 2 h of incubation in solutions with different pH; (**B**) EGCG release of EGCG/gelatin/γ-PGA nanoparticles in SGF and SIF at 37 °C for 0.5, 1, 2, 3, or 4 h.

The harsh environment of the gastrointestinal tract, including elevated pH and metabolic enzymes, is one of the factors that hamper EGCG oral bioavailability [2]. The stability of the produced EGCG/gelatin/γ-PGA nanoparticles was assessed over a range of pH values by resuspending them in buffer solutions. Figure 4A shows the release rates of EGCG from the nanoparticles after 2 h of incubation in different pH solutions. The release rate of EGCG gradually increased with increasing pH, being only 14.2% in the pH 3–4 solution but reaching 74.9% in the weak alkaline solution (pH 7.5). This may be attributed to the reduced positive charge exhibited by type-A gelatin in a weakly alkaline solution, leading to the disintegration of nanoparticles and massive release of EGCG. Furthermore, the EGCG release rate of EGCG/gelatin/γ-PGA nanoparticles was lower than that of EGCG/gelatin nanoparticles in acidic or neutral solutions but higher in weakly alkaline solutions. This may be due to the increased stability of the EGCG/gelatin/γ-PGA complex. These results indicate that EGCG/gelatin/γ-PGA nanoparticles are more stable in an acidic environment, offering better protection for EGCG.

To investigate the effect of proteases on the nanoparticles during digestion, the prepared EGCG/gelatin/γ-PGA nanoparticles were exposed to simulated gastric fluid (SGF) and simulated intestinal fluid (SIF) for four hours, and high-performance liquid chromatography (HPLC) was utilized to measure the release of EGCG at different time intervals (0.5, 1, 2, 3, and 4 h). As depicted in Figure 4B, EGCG/gelatin/γ-PGA nanoparticles remained stable in SGF, with a maximum EGCG release rate of 21.4% after four hours of incubation. Within the first 30 min, EGCG was rapidly released from the nanoparticles into the SIF at a rate that eventually reached 40%. The cumulative release rate of EGCG decreased slightly from 0.5–3 h, totaling 81%. From 3–4 h, the release rate of EGCG decreased sharply, and the cumulative release peaked at 84%. These results indicate that when exposed to pepsin in SGF, the nanoparticles remained relatively stable. However, in alkaline SIF, a large number of nanoparticles disintegrated, and EGCG was sustainably released. This can be attributed to the electrostatic interaction between gelatin and PGA, which hinders the contact between gelatin and protease. The high cumulative release rate suggests that EGCG/gelatin/γ-PGA nanoparticles serve as superior EGCG delivery carriers.

### 3.5. DPPH and ABTS Scavenging Activity of Nanoparticles

**Figure 5:**
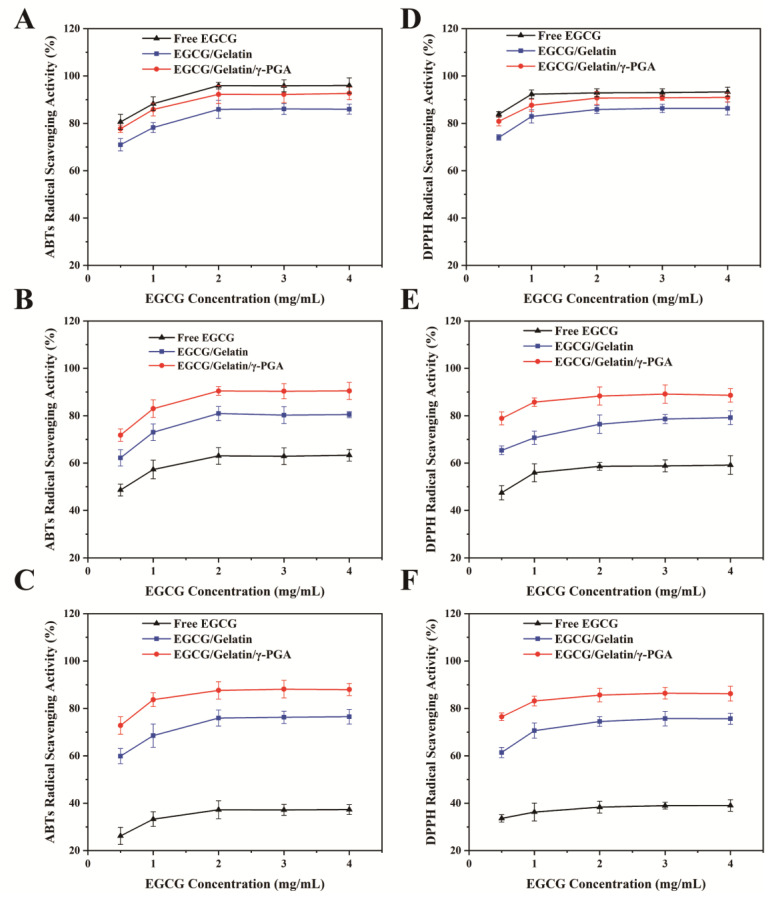
DPPH free radical scavenging activity of free EGCG, EGCG/gelatin nanoparticles, and EGCG/gelatin/γ-PGA nanoparticles kept at 25 °C for 0 (**A**), 7 (**B**), and 14 (**C**) days; ABTS free radical scavenging activity of free EGCG, EGCG/gelatin nanoparticles, and EGCG/gelatin/γ-PGA nanoparticles kept at 25 °C for 0 (**D**), 7 (**E**), and 14 (**F**) days.

The antioxidant activity of EGCG/gelatin and EGCG/gelatin/γ-PGA nanoparticles was compared to free EGCG using the DPPH and ABTS radical scavenging assays. As depicted in Figure 5, the antioxidant capacity of all samples increased with the concentration of EGCG, while that of both types of EGCG-embedded nanoparticles decreased slightly with increasing incubation time. In contrast, the antioxidant activity of free EGCG was significantly reduced.

On day 0 of storage, the DPPH radical scavenging activity of free EGCG was 93.3%, while that of EGCG/gelatin and EGCG/gelatin/γ-PGA nanoparticles were 86.3% and 90.7%, respectively (Figure 5A). As storage time progressed to day 7, the DPPH scavenging rate of free EGCG decreased significantly, falling below that of the two types of nanoparticles (Figure 5B). By day 14, the DPPH scavenging rate of EGCG/gelatin/γ-PGA nanoparticles was still maintained at 86.2%, which was higher than the 75.6% of EGCG/gelatin nanoparticles and the 39.1% of free EGCG (Figure 5C). The DPPH scavenging rate of free EGCG decreased by 2.4 times. These results suggest that the nanoparticles might shield EGCG from environmental oxygen oxidation during storage. Moreover, the EGCG/gelatin/γ-PGA nanoparticles had a greater antioxidant capacity than EGCG/gelatin nanoparticles throughout the storage period. This could be attributed to the more compact structure of EGCG/gelatin/γ-PGA nanoparticles, which reduced the contact of EGCG with oxygen.

Similarly, the ABTS radical-scavenging activities of all samples exhibited comparable trends (Figure 5C–E). After 14 days of storage, the ABTS scavenging rate of EGCG/gelatin/γ-PGA nanoparticles remained at 87.9%, higher than that of EGCG/gelatin nanoparticles (76.2%), while the ABTS scavenging rate of free EGCG decreased to 37.3% (Figure 5E). These findings imply that the EGCG was nearly fully preserved after being encapsulated in EGCG/gelatin/γ-PGA nanoparticles, indicating the protective effect of the nanoparticles.

### 3.6. EGCG Transport on Caco-2 Monolayers

The oral bioavailability of EGCG is reported to be very low, with only 0.1–1.1% of the administered dose reaching systemic circulation in human studies [46]. Generally, the bioactivity of EGCG is enhanced at concentrations higher than 10 and 20 mol/L. However, due to the significant biotransformation of EGCG in the intestine, the peak plasma concentration of EGCG in the human body is typically around 1 μmol/L [47]. As a result, improving the intestinal absorption of EGCG using nanoparticle carriers is highly significant.

**Figure 6:**
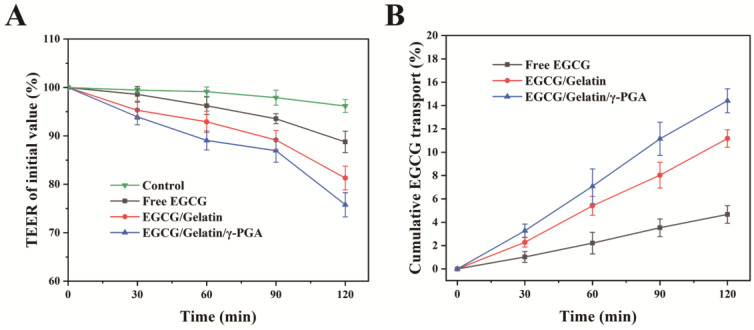
(**A**) TEER values of Caco-2 monolayers handled with free EGCG, EGCG/gelatin nanoparticles, and EGCG/gelatin/γ-PGA nanoparticles; (**B**) Cumulative EGCG transport through Caco-2 monolayers dealt with free EGCG, EGCG/gelatin nanoparticles, and EGCG/gelatin/γ-PGA nanoparticles. The pH of donor compartments was set at 6.2.

The TEER value is an indicator of the integrity of the Caco-2 monolayers, and a decrease in this value suggests a disruption in the tight monolayer connections. Before the transportation experiment, the Caco-2 monolayers had a suitable TEER value of 400–500 Ω × cm^2^ [48]. Figure 6A illustrates the impact of the nanoparticles on TEER during the experiment. The TEER percentage of the control group treated with HBSS buffer decreased to 96.2% of the initial value after 120 min of incubation. The TEER percentages of the Caco-2 monolayers treated with free EGCG, EGCG/gelatin nanoparticles, and EGCG/gelatin/γ-PGA nanoparticles decreased to 93.7%, 81.3%, and 75.8% of the initial value, respectively. The TEER values of the Caco-2 monolayers treated with nanoparticles decreased significantly compared to controls, and the EGCG/gelatin/γ-PGA nanoparticles had a greater impact on the TEER value. It has been found that the paracellular pathway may mediate macromolecular transport in Caco-2 monolayers [49]. When the tight junctions between Caco-2 cells open, molecules pass across the gap between the epithelial cells, causing the TEER value to decrease. Therefore, it appears that the EGCG/gelatin/γ-PGA nanoparticles may promote the penetration of EGCG through the paracellular pathway by opening the tight connections of Caco-2 monolayers.

The cumulative transport of EGCG through Caco-2 monolayers was investigated after incubation with EGCG, EGCG/gelatin nanoparticles, and EGCG/gelatin/γ-PGA nanoparticles for up to 120 min (Figure 6B). The amount of EGCG transported from the donor compartment to the receiver compartment gradually increased with incubation time. At 120 min, the cumulative transport rate with EGCG/gelatin/γ-PGA nanoparticles was 14.4%, which was higher than the rates of 4.7% for free EGCG and 11.2% for EGCG/gelatin nanoparticles. Nanoparticles smaller than 300 nm can pass through the cell membrane via clathrin-mediated endocytosis, which can increase the permeability of bioactive substances [50]. In this study, the permeability of EGCG was enhanced by embedding it in nanoparticles, likely due to the cellular absorption of the nanoparticles by the Caco-2 monolayers. Furthermore, compared to EGCG/gelatin nanoparticles, the cell transport rate of EGCG in EGCG/gelatin/γ-PGA nanoparticles was higher, possibly due to γ-PGA enhancing the cellular absorption of EGCG [23]. Co-administration of EGCG with γ-PGA has been shown to improve the therapeutic effect of EGCG on metabolism in obese type 2 diabetic mice [51]. Therefore, EGCG/gelatin/γ-PGA nanoparticles may better exert the health properties of EGCG, with potential applications in functional foods.

## 4. Conclusions

In this study, we present a novel self-assembly nanotechnology approach to prepare EGCG/gelatin/γ-PGA nanoparticles by simply mixing type-A gelatin with γ-PGA and EGCG in an aqueous solution. The EGCG/gelatin/γ-PGA nanoparticles had larger particle sizes than the EGCG/gelatin nanoparticles. Furthermore, the EGCG/gelatin/γ-PGA nanoparticles exhibited improved long-term storage stability and antioxidant activity of EGCG. These nanoparticles were found to be stable in SGF and released in SIF. Additionally, the TEER value and cumulative transport rate indicated that the EGCG/gelatin/γ-PGA nanoparticles enhanced the cell absorption of EGCG. Our findings suggest that EGCG/gelatin/γ-PGA nanoparticles could serve as an effective carrier for improving the stability and bioavailability of EGCG, which could have potential applications in the functional food industry. 

## Figures and Tables

**Table 1:** Mean particle sizes, PDI, and zeta potential of EGCG/gelatin/γ-PGA and gelatin/γ-PGA nanoparticles (*n* = 3 batches) ^a^ (*p* < 0.05).

(a) EGCG/gelatin/γ-PGA nanoparticles			
gelatin/ γ-PGA	Mean Particle Size (nm)	PDI	Zeta Potential (mV)
1:1	112.4 ± 4.4	0.475 ± 0.026	−24.3 ± 0.3
2:1	149.9 ± 5.3	0.341 ± 0.041	−22.2 ± 1.0
4:1	155.1 ± 7.3	0.585 ± 0.034	−23.9 ± 0.9
6:1	212.7 ± 6.5	0.564 ± 0.069	−19.2 ± 0.4
8:1	N/A ^b^	N/A	N/A
**(b) Gelatin/γ-PGA nanoparticles**			
1:1	104.2 ± 2.8	0.272 ± 0.035	−22.1 ± 0.6
2:1	116.9 ± 6.7	0.458 ± 0.053	−21.4 ± 0.3
4:1	118.1 ± 1.1	0.434 ± 0.062	−20.9 ± 0.2
6:1	N/A	N/A	N/A
8:1	N/A	N/A	N/A

^a^ The EGCG and γ-PGA concentrations were maintained at 1 and 1 mg/mL, respectively. ^b^ N/A: Aggregates were noticed precipitating.

## Data Availability

The data used to support the findings of this study can be made available by the corresponding author upon request.

## References

[1] Dai W., Ruan C., Zhang Y., Wang J., Han J., Shao Z., Sun Y., Liang J. (2020). Bioavailability enhancement of EGCG by structural modification and nano-delivery: A review. J. Funct. Foods.

[2] Yin Z., Zheng T., Ho C.-T., Huang Q., Wu Q., Zhang M. (2022). Improving the stability and bioavailability of tea polyphenols by encapsulations: A review. Food Sci. Hum. Wellness.

[3] Wen J.-J., Li M.-Z., Chen C.-H., Hong T., Yang J.-R., Huang X.-J., Geng F., Hu J.-L., Nie S.-P. (2022). Tea polyphenol and epigallocatechin gallate ameliorate hyperlipidemia via regulating liver metabolites and remodeling gut microbiota. Food Chem..

[4] Rady I., Mohamed H., Rady M., Siddiqui I.A., Mukhtar H. (2018). Cancer preventive and therapeutic effects of EGCG, the major polyphenol in green tea. Egypt. J. Basic Appl. Sci..

[5] Cao S.-Y., Zhao C.-N., Gan R.-Y., Xu X.-Y., Wei X.-L., Corke H., Atanasov A.G., Li H.-B. (2019). Effects and mechanisms of tea and its bioactive compounds for the prevention and treatment of cardiovascular diseases: An updated review. Antioxidants.

[6] Nikoo M., Regenstein J.M., Ahmadi Gavlighi H. (2018). Antioxidant and antimicrobial activities of (-)-epigallocatechin-3-gallate (EGCG) and its potential to preserve the quality and safety of foods. Compr. Rev. Food Sci. Food Saf..

[7] Barenys M., Gassmann K., Baksmeier C., Heinz S., Reverte I., Schmuck M., Temme T., Bendt F., Zschauer T.-C., Rockel T.D. (2017). Epigallocatechin gallate (EGCG) inhibits adhesion and migration of neural progenitor cells in vitro. Arch. Toxicol..

[8] Su Y.L., Leung L.K., Huang Y., Chen Z.-Y. (2003). Stability of tea theaflavins and catechins. Food Chem..

[9] Rein M.J., Renouf M., Cruz-Hernandez C., Actis-Goretta L., Thakkar S.K., da Silva Pinto M. (2013). Bioavailability of bioactive food compounds: A challenging journey to bioefficacy. Br. J. Clin. Pharmacol..

[10] Singh B.N., Shankar S., Srivastava R.K. (2011). Green tea catechin, epigallocatechin-3-gallate (EGCG): Mechanisms, perspectives and clinical applications. Biochem. Pharmacol..

[11] Peng Y., Meng Q., Zhou J., Chen B., Xi J., Long P., Zhang L., Hou R. (2018). Nanoemulsion delivery system of tea polyphenols enhanced the bioavailability of catechins in rats. Food Chem..

[12] Tang D.-W., Yu S.-H., Ho Y.-C., Huang B.-Q., Tsai G.-J., Hsieh H.-Y., Sung H.-W., Mi F.-L. (2013). Characterization of tea catechins-loaded nanoparticles prepared from chitosan and an edible polypeptide. Food Hydrocoll..

[13] Roger E., Lagarce F., Garcion E., Benoit J.-P. (2010). Biopharmaceutical parameters to consider in order to alter the fate of nanocarriers after oral delivery. Nanomedicine.

[14] Niu L., Li Z., Fan W., Zhong X., Peng M., Liu Z. (2022). Nano-strategies for enhancing the bioavailability of tea polyphenols: Preparation, applications, and challenges. Foods.

[15] Rashidinejad A., Boostani S., Babazadeh A., Rehman A., Rezaei A., Akbari-Alavijeh S., Shaddel R., Jafari S. (2021). Opportunities and challenges for the nanodelivery of green tea catechins in functional foods. Food Res. Int..

[16] Zhou X., Wu Y., Zhou X., Huang Z., Zhao L., Liu C. (2022). Elaboration of Cationic Soluble Soybean Polysaccharides-Epigallocatechin Gallate Nanoparticles with Sustained Antioxidant and Antimicrobial Activities. J. Agric. Food Chem..

[17] Bhushani J.A., Karthik P., Anandharamakrishnan C. (2016). Nanoemulsion based delivery system for improved bioaccessibility and Caco-2 cell monolayer permeability of green tea catechins. Food Hydrocoll..

[18] Jia Z., Dumont M.-J., Orsat V. (2016). Encapsulation of phenolic compounds present in plants using protein matrices. Food Biosci..

[19] Yan X., Zhang X., McClements D.J., Zou L., Liu X., Liu F. (2019). Co-encapsulation of epigallocatechin gallate (EGCG) and curcumin by two proteins-based nanoparticles: Role of EGCG. J. Agric. Food Chem..

[20] Evageliou V., Panagopoulou E., Mandala I. (2019). Encapsulation of EGCG and esterified EGCG derivatives in double emulsions containing whey protein isolate, bacterial cellulose and salt. Food Chem..

[21] Wang L., Chen S., Yu B. (2022). Poly-γ-glutamic acid: Recent achievements, diverse applications and future perspectives. Trends Food Sci. Technol..

[22] Wang R., Guo K., Zhang W., He Y., Yang K., Chen Q., Yang L., Di Z., Qiu J., Lei P. (2022). Poly-γ-Glutamic Acid Microgel-Encapsulated Probiotics with Gastric Acid Resistance and Smart Inflammatory Factor Targeted Delivery Performance to Ameliorate Colitis. Adv. Funct. Mater..

[23] Kashima N., Furuta K., Tanabe I. (2006). Permeability Enhancer.

[24] Tamura M., Hoshi C., Kimura Y., Suzuki T., Yamamoto-Maeda M. (2018). Effects of γ-polyglutamic acid on the cecal microbiota and visceral fat in KK-Ay/TaJcl male mice. Food Sci. Technol. Res..

[25] Lee E.-H., Son W.-C., Lee S.-E., Kim B.-H. (2013). Anti-obesity effects of poly-γ-glutamic acid with or without isoflavones on high-fat diet induced obese mice. Biosci. Biotechnol. Biochem..

[26] Sarika P., James N.R. (2016). Polyelectrolyte complex nanoparticles from cationised gelatin and sodium alginate for curcumin delivery. Carbohydr. Polym..

[27] Abedinia A., Nafchi A.M., Sharifi M., Ghalambor P., Oladzadabbasabadi N., Ariffin F., Huda N. (2020). Poultry gelatin: Characteristics, developments, challenges, and future outlooks as a sustainable alternative for mammalian gelatin. Trends Food Sci. Technol..

[28] Ranganathan S., Balagangadharan K., Selvamurugan N. (2019). Chitosan and gelatin-based electrospun fibers for bone tissue engineering. Int. J. Biol. Macromol..

[29] Eastoe J.E., Leach A.A., Ward A.G., Courts A. (1977). Chemical constitution of gelatin. The Science and Technology of Gelatin.

[30] Shutava T.G., Balkundi S.S., Vangala P., Steffan J.J., Bigelow R.L., Cardelli J.A., O’Neal D.P., Lvov Y.M. (2009). Layer-by-layer-coated gelatin nanoparticles as a vehicle for delivery of natural polyphenols. ACS Nano.

[31] Garcia J.P.D., Hsieh M.-F., Doma B.T., Peruelo D.C., Chen I.-H., Lee H.-M. (2013). Synthesis of gelatin-γ-polyglutamic acid-based hydrogel for the in vitro controlled release of epigallocatechin gallate (EGCG) from Camellia sinensis. Polymers.

[32] Ma X., Zhao S., Chen Y., Chen S. (2019). Preparation of Gamma Polyglutamic Acid (Γ-PGA)/Gelation Composite Nanoparticle and Application on Osmanthus Fragrance Slow-Release. Proc. IOP Conf. Ser. Mater. Sci. Eng..

[33] Chen Y.-C., Yu S.-H., Tsai G.-J., Tang D.-W., Mi F.-L., Peng Y.-P. (2010). Novel technology for the preparation of self-assembled catechin/gelatin nanoparticles and their characterization. J. Agric. Food Chem..

[34] Neilson A.P., Green R.J., Wood K.V., Ferruzzi M.G. (2006). High-throughput analysis of catechins and theaflavins by high performance liquid chromatography with diode array detection. J. Chromatogr..

[35] Zhang Y., Xu M., Hu C., Liu A., Chen J., Gu C., Zhang X., You C., Tong H., Wu M. (2019). Sargassum fusiforme fucoidan SP2 extends the lifespan of Drosophila melanogaster by upregulating the Nrf2-mediated antioxidant signaling pathway. Oxidative Med. Cell. Longev..

[36] Zhao P., Duan L., Guo L., Dou L.-L., Dong X., Zhou P., Li P., Liu E.-H. (2015). Chemical and biological comparison of the fruit extracts of Citrus wilsonii Tanaka and *Citrus medica* L.. Food Chem..

[37] Hubatsch I., Ragnarsson E.G., Artursson P. (2007). Determination of drug permeability and prediction of drug absorption in Caco-2 monolayers. Nat. Protoc..

[38] Muzolf M., Szymusiak H., Gliszczyńska-Świgło A., Rietjens I.M., Tyrakowska B.E. (2008). pH-dependent radical scavenging capacity of green tea catechins. J. Agric. Food Chem..

[39] Luo Z., Guo Y., Liu J., Qiu H., Zhao M., Zou W., Li S. (2016). Microbial synthesis of poly-γ-glutamic acid: Current progress, challenges, and future perspectives. Biotechnol. Biofuels.

[40] Hufschmid R., Teeman E., Mehdi B.L., Krishnan K.M., Browning N.D. (2019). Observing the colloidal stability of iron oxide nanoparticles in situ. Nanoscale.

[41] Song H., Wang Q., He A., Li S., Guan X., Hu Y., Feng S. (2022). Antioxidant activity, storage stability and in vitro release of epigallocatechin-3-gallate (EGCG) encapsulated in hordein nanoparticles. Food Chem..

[42] He A., Guan X., Song H., Li S., Huang K. (2020). Encapsulation of (−)-epigallocatechin-gallate (EGCG) in hordein nanoparticles. Food Biosci..

[43] Astete C.E., Sabliov C.M. (2006). Synthesis and characterization of PLGA nanoparticles. J. Biomater. Sci. Polym. Ed..

[44] Amani F., Rezaei A., Damavandi M.S., Doost A.S., Jafari S.M. (2022). Colloidal carriers of almond gum/gelatin coacervates for rosemary essential oil: Characterization and in-vitro cytotoxicity. Food Chem..

[45] You G., Niu G., Long H., Zhang C., Liu X. (2020). Elucidation of interactions between gelatin aggregates and hsian-tsao gum in aqueous solutions. Food Chem..

[46] Dube A., Nicolazzo J.A., Larson I. (2010). Chitosan nanoparticles enhance the intestinal absorption of the green tea catechins (+)-catechin and (−)-epigallocatechin gallate. Eur. J. Pharm. Sci..

[47] Hu B., Ting Y., Zeng X., Huang Q. (2012). Cellular uptake and cytotoxicity of chitosan–caseinophosphopeptides nanocomplexes loaded with epigallocatechin gallate. Carbohydr. Polym..

[48] Yang R., Liu Y., Gao Y., Yang Z., Zhao S., Wang Y., Blanchard C., Zhou Z. (2018). Nano-encapsulation of epigallocatechin gallate in the ferritin-chitosan double shells: Simulated digestion and absorption evaluation. Food Res. Int..

[49] Fu Q., Wang H., Xia M., Deng B., Shen H., Ji G., Li G., Xie Y. (2015). The effect of phytic acid on tight junctions in the human intestinal Caco-2 cell line and its mechanism. Eur. J. Pharm. Sci..

[50] Rejman J., Oberle V., Zuhorn I.S., Hoekstra D. (2004). Size-dependent internalization of particles via the pathways of clathrin-and caveolae-mediated endocytosis. Biochem. J..

[51] Bae K.-C., Park J.-H., Na A.-Y., Kim S.-J., Ahn S., Kim S.-P., Oh B.-C., Cho H.-C., Kim Y.W., Song D.-K. (2013). Effect of green tea extract/poly-γ-glutamic acid complex in obese type 2 diabetic mice. Diabetes Metab. J..

